# Dynamics of a national Omicron SARS-CoV-2 epidemic during January 2022 in England

**DOI:** 10.1038/s41467-022-32121-6

**Published:** 2022-08-03

**Authors:** Paul Elliott, Oliver Eales, Barbara Bodinier, David Tang, Haowei Wang, Jakob Jonnerby, David Haw, Joshua Elliott, Matthew Whitaker, Caroline E. Walters, Christina Atchison, Peter J. Diggle, Andrew J. Page, Alexander J. Trotter, Deborah Ashby, Wendy Barclay, Graham Taylor, Helen Ward, Ara Darzi, Graham S. Cooke, Marc Chadeau-Hyam, Christl A. Donnelly

**Affiliations:** 1 School of Public Health, Imperial College London, London, UK; 2 MRC Centre for Environment and Health, School of Public Health, Imperial College London, London, UK; 3 Imperial College Healthcare NHS Trust, London, UK; 4 National Institute for Health Research Imperial Biomedical Research Centre, London, UK; 5 Health Data Research (HDR) UK, Imperial College London, London, UK; 6 UK Dementia Research Institute, Imperial College London, London, UK; 7 MRC Centre for Global infectious Disease Analysis, London, UK; 8 Jameel Institute, Imperial College London, London, UK; 9 National Heart and Lung Institute, Imperial College Healthcare NHS Trust, London, UK; 10 Department of Infectious Disease, Imperial College London, London, UK; 11 CHICAS, Lancaster Medical School, Lancaster University, UK and Health Data Research, Lancaster, UK; 12 Quadram Institute, Norwich, UK; 13 Institute of Global Health Innovation, Imperial College London, London, UK; 14 Department of Statistics, University of Oxford, Oxford, UK

**Keywords:** Viral epidemiology, Epidemiology, SARS-CoV-2

## Abstract

Rapid transmission of the SARS-CoV-2 Omicron variant has led to record-breaking case incidence rates around the world. Since May 2020, the REal-time Assessment of Community Transmission-1 (REACT-1) study tracked the spread of SARS-CoV-2 infection in England through RT-PCR of self-administered throat and nose swabs from randomly-selected participants aged 5 years and over. In January 2022, we found an overall weighted prevalence of 4.41% (n = 102,174), three-fold higher than in November to December 2021; we sequenced 2,374 (99.2%) Omicron infections (19 BA.2), and only 19 (0.79%) Delta, with a growth rate advantage for BA.2 compared to BA.1 or BA.1.1. Prevalence was decreasing overall (reproduction number R = 0.95, 95% credible interval [CrI], 0.93, 0.97), but increasing in children aged 5 to 17 years (R = 1.13, 95% CrI, 1.09, 1.18). In England during January 2022, we observed unprecedented levels of SARS-CoV-2 infection, especially among children, driven by almost complete replacement of Delta by Omicron.

## Abstract (web summary)

The REACT-1 study measures the community prevalence of SARS-CoV-2 in England through repeated cross-sectional surveys. Here, the authors present data from REACT-1 that document the increase in infection prevalence, particularly among children, associated with the Omicron variant in January 2022.

## Introduction

November 2021 saw the identification of the Omicron variant in Botswana and South Africa, its rapid replacement of the Delta variant within South Africa^1^, and on 26 November, the classification by WHO of Omicron as a variant of concern^2^. By December 1, 2021, Omicron had been identified in the UK^3^ and the USA^4^ as well as other countries, including Belgium, Hong Kong and Israel, initially in travel-related cases.

By mid- to late December 2021, Omicron had become the dominant variant in the UK^5–7^ and had been detected in most European countries and US states. Not only was the increase in Omicron in England^5^ and elsewhere^8^ extremely rapid, but the rate of replacement of Delta by Omicron was over three-times higher than that of Alpha by Delta^5^. In some countries, social distancing policies were brought back into force^9^ and vaccination programmes accelerated^10^, while some healthcare systems struggled to cope with the associated increased healthcare demands^11,12^.

The REal-time Assessment of Community Transmission-1 (REACT-1) study tracked the spread of the SARS-CoV-2 virus among randomly-selected community samples in England, approximately monthly since May 2020, avoiding the biases associated with case incidence data and the delays inherent in hospitalisations and deaths^13^. We document the transmission dynamics of SARS-CoV-2 in England during January 2022 (round 17) following the emergence of Omicron as the dominant variant during December 2021^5,7^.

## Results

### Prevalence and sequencing results

**Fig. 1:**
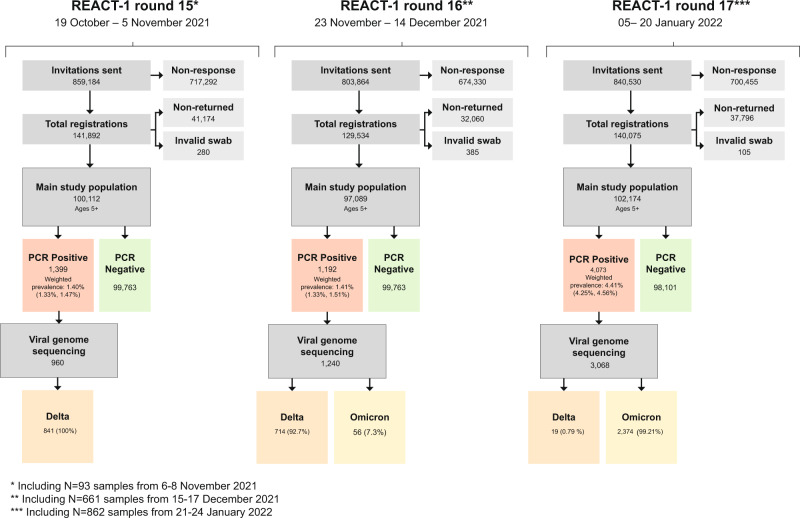
Study overview. Flow chart showing numbers of participants in round 15 (October 19–November 5, 2021), round 16 (November 23–December 14, 2021) and round 17 (January 5–20, 2022) of REACT-1.

In round 17 (5 to 20 January 2022) we invited 840,530 participants of whom 102,174 (12.2%) registered and returned a throat and nasal swab providing a valid SARS-CoV-2 RT-PCR test result (Fig. 1). The characteristics of individuals who took part in the study were broadly similar to those of the target population we invited, although in both rounds 16 (23 November to 14 December 2021) and 17, responders were slightly older and from more affluent areas than the general population (Supplementary Fig. S1). We found 4,073 positive swabs (based on detection of N gene with cycle threshold (Ct) value below 37 or both N and E gene detected) yielding a weighted prevalence of 4.41% (95% CrI, 4.25%, 4.56%). This was the highest prevalence observed in REACT-1 since the start of data collection in May 2020 (Supplementary Table S1) and over three-fold higher than in round 16. At the same time, in round 17, more participants reported protective behaviours with 25.7% (95% confidence interval [CI], 25.4%, 25.9%) shielding and 48.7% (95% CI, 48.4%, 49.0%) always wearing a mask indoors, compared to round 16, with 9.2% (95% CI, 9.0%, 9.4%) and 33.0% (95% CI, 32.7%, 33.3%) respectively.

**Fig. 2:**
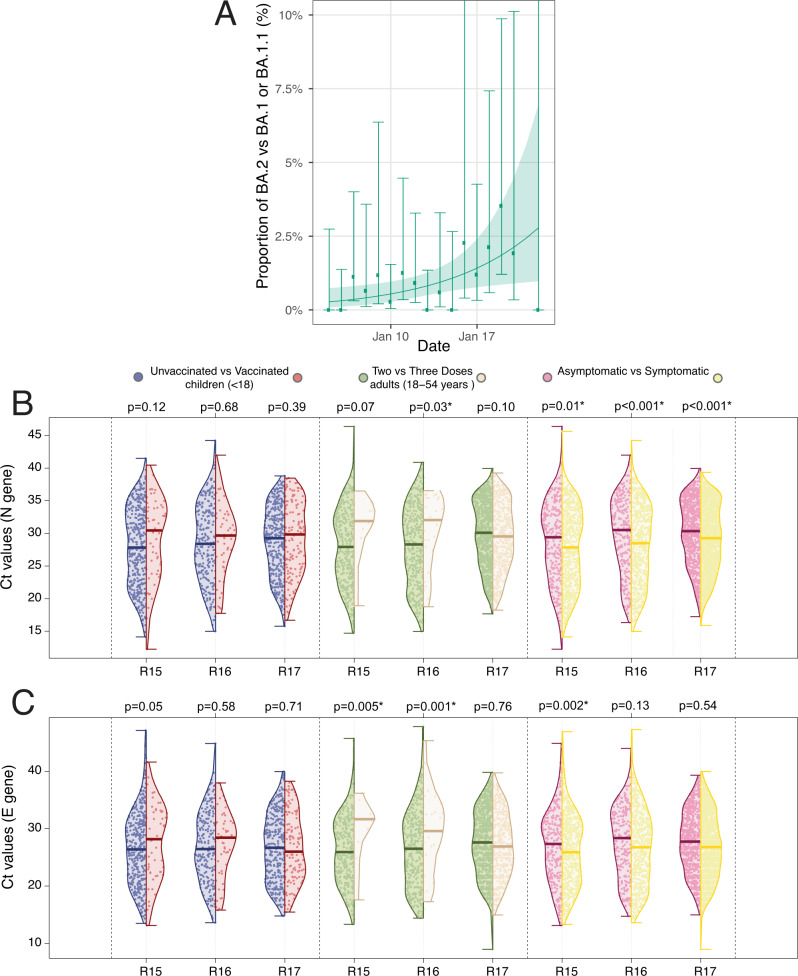
Description of the Omicron positive swabs. Daily proportion of BA.2 (vs BA.1 or BA.1.1) infections among (*n* = 2393) positive swabs with determined lineage and at least 50% genome coverage in round 17 (**A**). Point estimates (means) are represented (dots) along with 95% confidence intervals (vertical lines). Smoothed estimates of the proportion are also shown (solid line) together with their 95% credible intervals (shaded regions). No sequencing data were available for January 21, 2022. Distribution of the Ct value for the N gene (**B**) and E gene (**C**) in swab-positive samples from round 15 (Delta), round 16 (predominantly Delta) and round 17 (predominantly Omicron). Within each round, distributions are compared (i) for vaccinated vs. unvaccinated participants aged 17 years and under, (ii) those having received three vs two vaccine doses in adults aged 18 to 54 years, and (iii) for those reporting any symptoms vs those not reporting any symptom in the month prior to swabbing. For each comparison, we report the P-value from a non-parametric (Kruskal-Wallis) test.

We sequenced samples from 3,028 positive swabs from round 17 resulting in 2,393 determined viral genomes (Table 1). Of these 2,374 (99.2%, 95% CI, 98.8%, 99.5%) were Omicron and 19 (0.79%, 95% CI, 0.48%, 1.24%) Delta or Delta sub-lineages. Of the 2,374 Omicron lineages 13 (0.55%, 95% CI, 0.29%, 0.93%) were B.1.1.529, 1,822 (76.8%, 95% CI, 75.0%, 78.4%) BA.1, 519 (21.9%, 95% CI 20.2%, 23.6%) BA.1.1, 19 (0.80%, 95% CI, 0.48%, 1.25%) were BA.2, and 1 (0.04% 95% CI 0.00%, 0.23%) was BA.3 (obtained on January 17, 2022). We estimated a daily growth rate advantage of 0.14 (95% CrI 0.03, 0.28) in the odds of BA.2 (vs BA.1 or BA.1.1) corresponding to an additive R advantage of 0.46 (95% CrI, 0.10, 0.92), with the proportion of BA.2 estimated at 2.84% (95% CrI, 0.97%, 6.98%) on January 20, 2022 (Fig. 2A).

**Table 1:** Proportion of each of the *N* = 2393 SARS-CoV-2 lineage detected in positive samples with at least 50% genome coverage from round 17

Sub-lineage	*N* (2393)	Percentage (95% CI)	
**Delta/Delta sub-lineages**			
AY.34.1	1	0.04%	(0.01%, 0.24%)
AY.36	1	0.04%	(0.01%, 0.24%)
AY.4	7	0.29%	(0.14%, 0.60%)
AY.4.2.1	1	0.04%	(0.01%, 0.24%)
AY.4.2.2	2	0.08%	(0.02%, 0.30%)
AY.4.3	1	0.04%	(0.01%, 0.24%)
AY.5	1	0.04%	(0.01%, 0.24%)
AY.75	1	0.04%	(0.01%, 0.24%)
AY.98.1	1	0.04%	(0.01%, 0.24%)
B.1.1.174	1	0.04%	(0.01%, 0.24%)
B.1.617.2	2	0.08%	(0.02%, 0.30%)
**Omicron/Omicron sublineages**			
B.1.1.529	13	0.54%	(0.32%, 0.93%)
BA.1	1,822	76.14%	(74.39%, 77.80%)
BA.1.1	519	21.69%	(20.08%, 23.38%)
BA.2	19	0.79%	(0.51%, 1.24%)
BA.3	1	0.04%	(0.01%, 0.24%)

Results are based on 3,028 positive sequenced samples. We report the point estimate of the proportion and the 95% confidence interval in parentheses.

Ct values of swab-positive adults aged 18 to 54 years having received a third vaccine dose (compared to those having received two vaccine doses) were higher (lower viral load) in round 16 for N and E gene (when Delta predominated), and for E gene in round 15 (October 19 to November 5, 2021, all Delta), but not in round 17 (predominantly Omicron), suggesting less protection from infection by the booster dose for Omicron than for Delta. Irrespective of the round, we found lower Ct values (higher viral load) for N gene in swab-positives reporting symptoms compared to those not reporting any symptoms (*p* < 0.01) (Fig. 2B, C).

### Temporal trends

**Fig. 3:**
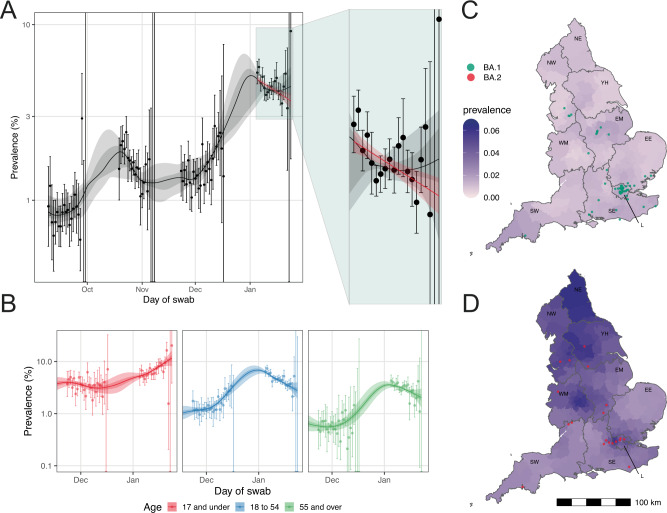
Temporal and geographical distribution of SARS-CoV-2 swab positivity. Comparison of an exponential model fit to SARS-CoV-2 swab-positivity data in round 17 (*n* = 102,174 participants with valid RT-PCR test results, in red), and a P-spline model fit to all rounds of REACT-1 (black, shown here only for rounds 14, 15, 16 and 17) (**A**). The solid red line represents the posterior mean estimate from the exponential growth model, and the shaded red region the 95% posterior credible interval for the exponential models. The solid black line represents the posterior mean smoothed estimate of the weighted prevalence and the shaded grey region shows 50% (dark grey) and 95% (light grey) posterior credible interval for the P-spline model. Results are presented for each day (X axis) of sampling for round 14, round 15, round 16 and round 17 and the weighted prevalence of swab-positivity is shown (Y axis) on a log scale. Weighted observations (black dots) and their 95% credible intervals (vertical lines) are also shown. Results from similar P-spline models for those aged 17 years and under (*n* = 10,638 participants with valid RT-PCR test results, in red), those aged 18 to 54 years inclusive (*n* = 39,676 participants with valid RT-PCR test results, in blue) and those aged 55 years and over (*n* = 51,860 participants with valid RT-PCR test results, in green) (**B**). Results are presented for round 16 and 17. Solid lines represent the posterior mean estimate of the smoothed weighted prevalence, and shaded regions represent the 50% (dark color) and 95% (light color) posterior credible interval of the smoothed prevalence. Weighted observations (dots) and 95% credible intervals (vertical lines) are also represented. Neighbourhood smoothed average SARS-CoV-2 swab-positivity prevalence by lower-tier local authority area for round 16 (**C**) and round 17 (**D**) in England. Neighbourhood prevalence calculated from nearest neighbours (the median number of neighbours within 30 km in the study). We represent the (jittered) location where we first detected BA.1 and sub-lineages (*N* = 56) in round 16 (**C**) and BA.2 and sub-lineages (*N* = 19) in round 17 (**D**). Regions: NE North East, NW North West, YH Yorkshire and The Humber, EM East Midlands, WM West Midlands, EE East of England, L London, SE South East, SW South West.

We show a substantial increase in weighted prevalence between round 16 (November 23 to December 14, 2021) and round 17 (January 5 to 20, 2022) (Supplementary Table S1, Fig. 3A), followed by a within-round decline overall (*R* = 0.95, 95% CrI, 0.93, 0.97, Table S2).

However, analyses stratified by age group showed a within-round 17 increasing weighted prevalence in those aged 17 years and under (Fig. 3B) and estimated within-round R of 1.13 (95% CrI, 1.09, 1.18, Supplementary Table S2) in that age group.

At all ages, weighted prevalence increased from two- (in those aged 5 to 11 years) to almost twelve-fold (in those aged 75 years and over) between round 16 and round 17 (Supplementary Table S3A, Supplementary Fig. S2). The highest weighted prevalence in round 17 was observed in those aged 5 to 11 years at 7.85% (95% CrI, 7.10%, 8.69%) and the lowest in those aged 75 years and over at 2.46% (95% CrI, 2.16%, 2.80%). Weighted prevalence also increased between rounds in all regions, with local areas of high prevalence detected (Supplementary Table S3A, Fig. 3C, D, Supplementary Fig. S3). The (*N* = 56) swab-positive participants with Omicron (BA.1 sub-lineage) detected in round 16 and (*N* = 19) swab-positive participants with BA.2 Omicron sub-lineages in round 17 were mainly found in London (Fig. 3C, D).

### Risk factors: demographic, symptom-related and exposure-related

Our results are suggestive of within-household transmission with weighted prevalence increasing with (i) size of household from 3.15% (95% CrI, 2.86%, 3.48%) in single-person households to 7.72% (95% CrI, 6.47%, 9.18%) in households with 6 or more persons and (ii) number of children in the household from 3.57% (95% CrI, 3.41%, 3.74%) in households without children to 5.92% (95% CrI, 5.60%, 6.25%) in households with one or more children (Supplementary Table S3B).

We found highest weighted prevalence among those having been in contact with a confirmed (12.8%, 95% CrI, 12.2%, 13.5%) or a suspected COVID-19 case (9.38%, 95% CrI, 8.14%, 10.8%), and in those reporting classic COVID-19 symptoms (loss or change of sense of smell or taste, fever, new persistent cough) in the month prior to swabbing at 15.9% (95% CrI, 15.1%, 16.7%).

Multivariable logistic regression models identified variables independently associated with higher risk of swab-positivity, including (i) living in a large household with ORs of 1.28 (95% CI 1.17, 1.40) and 1.73 (95% CI 1.44, 2.08) for households of 3 to 5 and 6 or more persons, respectively, compared to single-person households; (ii) living in urban (vs. rural) areas with OR of 1.24 (95% CI 1.13, 1.35), and in the most vs least deprived areas with OR of 1.34 (95% CI 1.20, 1.49) (Table 2). We also found higher risk of swab-positivity in essential/key worker (other than a healthcare or care home worker) with mutually adjusted Odds Ratio (OR) of 1.15 (95% CI, 1.05, 1.26). On average, non-healthcare essential workers reported 7.56 (95% CI, 6.62, 8.49) contacts in the day before swabbing (excluding contacts with household members), which is higher than the 4.52 (95% CI, 4.18, 4.87) contacts in healthcare/care home workers; 2.92 (95% CI, 2.77, 3.07) in other workers; 3.60 (95% CI 0.63, 6.57) in the unemployed; and 3.97 (95% CI, 3.34, 4.60) in those with unknown occupation.

**Table 2:** Multivariable logistic regression for SARS-CoV-2 swab-positivity in round 17

Variable		Unadjusted	Adjusted for age and sex	Mutually adjusted*
Gender	Male	Ref	Ref	Ref
	Female	0.94 (0.88, 1.00)	0.92 (0.86, 0.98)	0.93 (0.87, 0.99)
Employment type	Health care or care home worker	1.16 (1.03, 1.30)	1.12 (1.00, 1.26)	1.06 (0.94, 1.19)
	Other essential/key worker	1.24 (1.14, 1.36)	1.20 (1.09, 1.31)	1.15 (1.05, 1.26)
	Other worker	Ref	Ref	Ref
	Not full-time, part-time, or self-employed	0.77 (0.71, 0.83)	0.92 (0.84, 1.00)	0.89 (0.82, 0.97)
	Unknown	1.34 (1.10, 1.64)	1.22 (0.98, 1.50)	1.12 (0.91, 1.39)
Ethnic group	White	Ref	Ref	Ref
	Asian	1.47 (1.30, 1.67)	1.25 (1.10, 1.41)	1.11 (0.98, 1.26)
	Black	1.63 (1.34, 1.99)	1.42 (1.16, 1.74)	1.18 (0.96, 1.45)
	Mixed	1.39 (1.12, 1.72)	1.09 (0.88, 1.36)	1.07 (0.86, 1.33)
	Other	1.62 (1.24, 2.11)	1.44 (1.10, 1.88)	1.30 (0.99, 1.71)
	Unknown	1.03 (0.84, 1.26)	1.07 (0.87, 1.31)	1.01 (0.82, 1.24)
Household size	1-2	Ref	Ref	Ref
	3-5	1.61 (1.51, 1.71)	1.31 (1.22, 1.42)	1.28 (1.17, 1.40)
	6+	2.40 (2.04, 2.83)	1.89 (1.59, 2.24)	1.73 (1.44, 2.08)
Number of children in the household	0	Ref	Ref	Ref
	1+	1.66 (1.55, 1.77)	1.30 (1.19, 1.41)	1.11 (1.01, 1.24)
	Unknown	1.33 (1.16, 1.53)	1.19 (0.98, 1.46)	1.12 (0.91, 1.38)
Living in urban area	Yes	1.47 (1.35, 1.60)	1.39 (1.27, 1.51)	1.24 (1.13, 1.35)
	No	Ref	Ref	Ref
	Unknown	1.70 (0.90, 3.23)	1.42 (0.75, 2.70)	1.33 (0.70, 2.53)
Deprivation	1 Most deprived	1.70 (1.54, 1.89)	1.61 (1.45, 1.79)	1.34 (1.20, 1.49)
	2	1.31 (1.18, 1.44)	1.26 (1.14, 1.39)	1.18 (1.06, 1.30)
	3	1.14 (1.03, 1.25)	1.13 (1.03, 1.25)	1.12 (1.02, 1.24)
	4	1.07 (0.97, 1.17)	1.07 (0.98, 1.18)	1.06 (0.97, 1.17)
	5 Least deprived	Ref	Ref	Ref

^*^Odds ratios are mutually adjusted for all variables shown and for age and region.

Results are presented as unadjusted Odds Ratios (95% confidence interval), adjusted for age and sex and additionally, for region and all other variables (mutually adjusted OR).

### Linking prevalence and national severe outcome incidence

Matching the daily prevalence of swab-positivity in REACT-1 to publicly available data on hospitalisations we estimated lag time of 19 (95% CrI 18, 20) days for rounds 1 to 12 (May 1, 2020 to May 20, 2021) and 16 (95% CrI 14, 18) days for rounds 13 (June 24 to July 12, 2021) to 16. We observed high consistency between daily prevalence and the (lagged) hospitalisations data from 15 June to 30 November 2021, but from 1 December onwards (when Omicron variant began to emerge in REACT-1 data), the 16 days lag may appear too long. This may to some extent reflect incidental hospitalisations with Omicron due to its high population prevalence and support using an Omicron-specific lag time, but data were too sparse in the present study to be able to estimate such a lag with suitable precision.

**Fig. 4:**
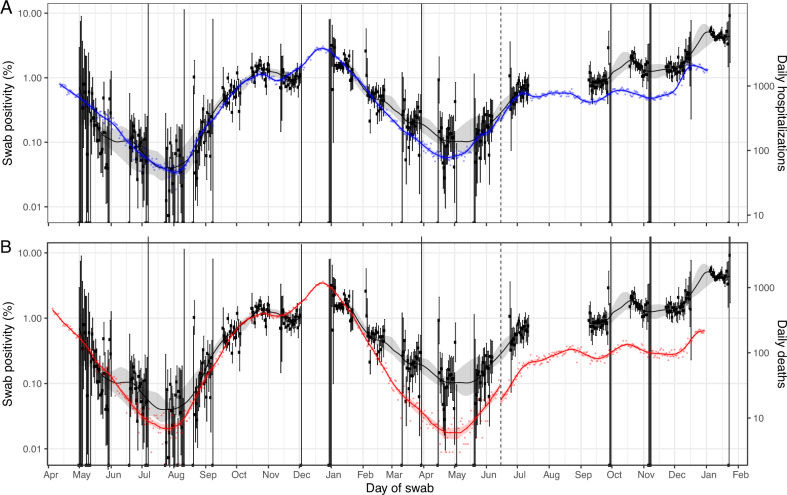
Comparison of COVID-19 daily hospitalisations and deaths with SARS-CoV-2 swab-positivity as measured in REACT-1. Daily swab-positivity for all 17 rounds of the REACT-1 study (black points with 95% confidence intervals, left-hand y-axis) with P-spline estimates for swab-positivity (solid black line, shaded area is 95% confidence interval). **A** Daily hospitalisations in England (blue points, right-hand y-axis) and P-spline model estimates for expected daily hospitalisations in England (solid blue line, shaded area is 95% confidence interval, right-hand y-axis). Daily hospitalisations have been shifted by 19 days (95% CI, 18, 20) backwards in time along the x-axis up to June 14, 2021 and by 16 days (95% 14, 18) thereafter. **B** Daily deaths in England (red points, right-hand y-axis) and P-spline model estimates for expected daily deaths in England (solid red line, shaded area is 95% confidence interval, right-hand y-axis). Daily deaths have been shifted by 26 days (95% CI, 25, 26) backwards in time along the x-axis up to June 14, 2021 and by 16 days (95% CI, 15, 17) thereafter. The scaling parameter estimates were 0.060 (0.058, 0.062) and 0.24 (0.23, 0.25) for deaths and hospitalisations, respectively.

Our results also showed: a close correspondence of prevalence of swab-positivity with hospitalisation (shifted by the estimated lag) through round 8 (January 6 to 22, 2021); an apparent reduced risk of hospitalisation vs. prevalence through rounds 9 to 11 (February 4 to May 3, 2021); the two coming together again in rounds 12 and 13 (May 20 to July 12, 2021) as Delta replaced Alpha; a further period of reduced risk of hospitalisation during rounds 14 to 15 (September 9 to November 5, 2021); and finally a further coming together in December 2021 as Omicron took off, followed by a rapid divergence once again (Fig. 4). Time lag estimates for death were 26 (95% CrI 25, 26) days for rounds 1-12 and 16 (95% CrI 15, 17) days for round 13-15 and trends showed a marked and consistent reduction in risk of death compared to prevalence of swab-positivity throughout rounds 9 to 16 (February 4 to December 14, 2021).

## Discussion

The Omicron epidemic in January 2022 was further advanced in England than in the USA and most European countries. During December 2021 Omicron almost completely replaced Delta in England^5–7^, with the estimated peak in prevalence around six weeks after Omicron was first identified in England. Although the BA.1 sub-lineage still dominated, our data during January 2022 show an increase in the proportion of daily infections from BA.2 compared to BA.1 and its sub-lineage BA.1.1 with an R advantage of 0.46. Similar data showing a transmission advantage of BA.2 compared to BA.1 were reported from the national routine testing data in the UK^14^ and in Denmark^15^.

As a result of the rapid rise in Omicron infections, during round 17 (January 2022), we saw the highest prevalence of SARS-CoV-2 infections ever observed in the REACT-1 study to that time. Overall prevalence was nearly three-fold higher than at the peak of the second wave in January 2021, with a near 12-fold increase in the oldest age group (75 years and over) since round 16 (November to December 2021). However, the dynamics underlying these population-level trends are complex with the prevalence decreasing in adults but increasing in children through January 2022. This is likely the consequence of the peak occurring during the end-of-year school break, causing a delay to school-based transmission among children. Also the high rates observed in children during round 16 were predominantly driven by Delta^5^, whereas round 17 trends were overwhelmingly due to Omicron infections. Essential/key workers other than healthcare/care home workers were found to be at increased risk of infection, even when other risk factors were adjusted for. Importantly, this group reported, on average, more contacts in the day before swabbing than other groups. How rates of contact have changed for different risk groups over the course of the pandemic warrants further investigation.

An estimated 20 to 40 times greater antibody titre is required for neutralization of Omicron than for Delta^16^, although individuals who had received a third vaccine dose did show increased neutralisation of the Omicron variant^17,18^. Among infected individuals in round 17, the distribution of Ct values was similar in adults who had received three compared to two doses of vaccine, suggesting that the booster did not affect viral load for Omicron.

These findings notwithstanding, crucially, booster doses remain highly effective at reducing the risks of severe disease^3,19,20^. Our comparison of infection prevalence data from REACT-1 and public data on hospitalisations and deaths indicate that although trends in these serious outcomes continue to track infections (albeit with a time lag), this is at a lower level than in previous waves, before widespread rollout of vaccination in England (from January 2021) and greater access to effective therapeutics for severe disease (such as corticosteroid and other immunomodulatory therapy)^21^. Nonetheless, it will be important to continue to monitor hospitalisations and deaths closely in view of the high levels of infection, including among the older population, and as restrictions were lifted in England and elsewhere^22,23^.

Our study has limitations. In round 17, 12.2% of the invited participants returned swabs producing valid RT-PCR test results. Our descriptive analyses showed that those who agreed to participate were slightly older and from more affluent areas than the general population. We use weights, calculated for each participant in each round, to adjust for differential response rates in calculating prevalence estimates, but these corrections may not fully eliminate all biases. As implemented in subsequent rounds of REACT-1 incentives can improve the response rate in the younger and less affluent populations^24^. Changes in the way the swab samples were transported and tested may have introduced small changes in results across rounds, although these should not have affected within-round trends. Our results comparing trends in infections with those in hospitalisations and deaths suggest a divergence in the most recent data consistent with reduced severity of Omicron. However, we were unable to assess in our data to what extent this reflects the fact that the Omicron wave occurred at a time of high levels of immunity in the population as a result of both natural infection and the successful rollout of the vaccination programme in England^25^.

In conclusion, we have shown a substantial and rapid rise in infections from early December 2021 through January 2022 as the Omicron variant took hold and almost completely replaced Delta in England. Among school-aged children there was a rise in prevalence as they returned to school in January after the end-of-year break^14,19,20^. Many countries relaxed restrictions as the peak in Omicron cases passed, including in England where the requirement to self-isolate if testing positive for COVID-19^26^ was removed despite the high prevalence of SARS-CoV-2 infection following contact with a known case. While vaccination (including the booster campaign) remains the mainstay of the defence against SARS-CoV-2 given the high levels of protection against severe disease^14,19,20^, further measures beyond vaccination may be required in the future in the event of the emergence of a new variant with greater potential than Omicron for hospitalisations and deaths.

## Methods

### Study design

The REACT-1 study involves a series of cross-sectional surveys of random samples of the population of England at ages 5 years and over^27^, conducted approximately monthly since May 2020. Round 17 (January 5 to 20, 2022) included 102,174 participants with a valid self-administered throat and nose swab test result for SARS-CoV-2 by reverse transcription polymerase chain reaction (RT-PCR) (including 862 samples obtained between January 21 and 24, 2022). Up to round 13 (June 24 to July 12, 2021), we collected dry swabs sent by courier to the laboratory on a cold chain but from round 14 (September 9 to 27, 2021 including 509 samples from 28-30 September) we switched to ‘wet’ (saline) swabs which (round 14) were sent to the laboratory either by courier (no cold chain) or priority post, and from round 15 (October 19 to November 5, 2021) onwards by priority post only. From round 16, we included a multiplex for detection of influenza A and B as well as SARS-CoV-2 (only the SARS-CoV-2 results are presented here). Because of delays in the post for return of swabs, we include a small proportion of samples obtained after the nominated closing date for each round of the study from round 14.

The sampling frame was the general practitioner list of patients in England held by the National Health Service. Participants registered and completed a questionnaire, providing information on age, sex, residential postcode, ethnicity, household size, occupation, potential contact with a COVID-19 case, symptoms and other variables^28^. We used the residential postcode to link to an area-level Index of Multiple Deprivation^29^ and urban/rural status^30^.

A positive test result was recorded if both N gene and E gene targets were detected or if N gene was detected with Ct value below 37. Initially we aimed to obtain approximately equal numbers of participants in each lower-tier local authority (LTLA) in England (*N* = 315), but from round 12 (May 20 to June 7, 2021) we switched to obtaining a random sample in proportion to population size at LTLA level. We compare results for SARS-CoV-2 with those obtained during October to December 2021 in round 15 (*N* = 100,112)^31^ and round 16 (*N* = 97,089)^5^.

Samples testing positive with Ct 34 or less in either the E or N gene were sent for viral genome sequencing to the Quadram Institute, Norwich, UK. We used the ARTIC protocol^32^ (version 4) for viral RNA amplification, CoronaHiT for preparation of sequencing libraries^33^, the ARTIC bioinformatics pipeline^32^ and assigned lineages using PangoLEARN (version 2022-01-20)^34^.

### Data analyses

We estimated round-specific weighted prevalence using random iterative method (rim) weighting^35^ to provide prevalence estimates for the population of England as a whole, and by region, adjusting for age, sex, deciles of the Index of Multiple Deprivation^29^, LTLA counts, and ethnic group and 95% credible intervals overall and for sub-groups. For round 17 we fit a Bayesian logistic regression model to the proportion of BA.2 lineage compared to the BA.1 lineage (or its sub-lineage BA.1.1) to estimate daily growth rate advantage for the odds of BA.2 versus BA.1. We then estimated the additive reproduction number (R) advantage as the product of the daily growth rate advantage and the Omicron-specific mean generation time^36^.

We used an exponential model of growth/decay to examine temporal trends in swab-positivity assuming a binomial distribution for the proportion of positives by day of swabbing where reported (or day of first Post Office scan if available) using a bivariate No-U-Turn Sampler and a uniform prior distribution for the probability of swab-positivity^37^. R was estimated assuming a gamma-distributed generation time with Omicron-specific mean 3.3 days and standard deviation 3.5 days (shape *n* = 0.89 and rate *β* = 0.27) as^36^:

1 $$R={\left(1+\frac{r}{\beta }\right)}^{n}$$

where *r* is the daily exponential growth/decay rate.

To visualise temporal trends in swab-positivity, we used a No-U-Turn Sampler in logit space to fit a Bayesian penalised-spline (P-spline) model^38^ to the daily data, split into approximately 5-day sections by regularly spaced knots. Edge effects were minimised by adding further knots beyond the study period. We used fourth-order basis splines (b-splines) over the knots, including a second-order random-walk prior distribution on the coefficients of the b-splines to guard against overfitting; the prior penalised against changes in growth rate unless supported by the data^39^. We also fit age-group-specific P-splines separately with the smoothing parameter obtained from the model fit to all the data.

We used a neighbourhood spatial smoothing method to examine geographical variation in SARS-CoV-2 prevalence at the LTLA level. For each of 15 randomly selected participants within an LTLA, we calculated the prevalence of infection among the nearest M people, where M was the median number of study participants within 30 km, and then estimated the smoothed neighbourhood prevalence in that area.

We compared (using a Kruskal–Wallis test) Ct values as a proxy for viral load among test-positive swabs (N gene and E gene where Ct>0), by vaccination status (lagged by a 14-day period from date of vaccination) and symptom status across rounds 15 to 17, where information on vaccination history and dates of vaccination was obtained (with consent) by linking to data from the national COVID-19 vaccination programme.

We used logistic regression to estimate the odds of testing positive by employment, ethnicity, household size, children in household, urban area, and deprivation, adjusting for age, region and the other variables examined. We estimated the average number of contacts in the day before swabbing (excluding contacts with household members) by employment type to aid interpretation of the logistic regression results.

We compared daily swab-positivity prevalence in REACT-1 with daily hospitalisations and (separately) deaths from (external) national data. We first fit P-spline models as described above to the daily hospital admissions and to the daily death data. We then fit a model to match daily weighted prevalence, and smoothed daily hospitalisation/death. This model included (i) a time lag parameter corresponding to the time between the P-spline estimate for hospitalisations or deaths and daily prevalence, and (ii) a scaling parameter, corresponding to the percentage of swab-positive participants with (lagged) hospitalisations or deaths. Estimation was done using in-house R scripts maximising the binomial likelihood based on daily weighted prevalence. The scaling parameter was estimated using REACT-1 data up to and including round 7 (November 13 to December 3, 2020), before the vaccination programme in England began and was kept constant thereafter. To account for variant-specific lags, we considered the original lag estimate (based on rounds 1 to 7) for round 1 (May 1 to June 1, 2020) to round 12 (May 20 to June 7, 2021) when Alpha dominated in England and a second lag parameter thereafter, estimated from round 13 (June 24 to July 12, 2021) to round 16 (November 23 to December 14, 2021), when Delta dominated. The second lag parameter was estimated fixing the scaling parameter to the estimate obtained based on analysis of data from rounds 1 to 7.

Analyses were performed with R software, version 4.0.5.

### Ethics

We obtained research ethics approval from the South Central-Berkshire B Research

Ethics Committee (IRAS ID: 283787). Participants in the study (or their parents or guardians for children) provided informed consent.

### Public involvement

A Public Advisory Panel provides input into the design, conduct, and dissemination of the REACT research program.

### Reporting summary

Further information on research design is available in the Nature Research Reporting Summary linked to this article.

## Supplementary information

Supplementary Information

Description of Additional Supplementary Files

Supplementary Data 1

Reporting Summary

## Data Availability

Access to REACT-1 individual-level data is restricted to protect participants’ anonymity. Summary statistics, descriptive tables, from the current REACT-1 study are available at 10.5281/zenodo.6819880. REACT-1 study materials are available for each round at https://www.imperial.ac.uk/medicine/research-and-impact/groups/react-study/react-1-study-materials/. Sequence read data are available without restriction from the European Nucleotide Archive at https://www.ebi.ac.uk/ena/browser/view/PRJEB37886, and consensus genome sequences are available from the Global initiative on sharing all influenza data (GISAID). Accession numbers for these data are available in Supplementary Data 1.

## Code availability

Code used to generate the present results are available at https://github.com/mrc-ide/reactidd/tree/master/R/r17_scripts (10.5281/zenodo.6819880).
