# Supplementary material for: Dynamics of a national Omicron SARS-CoV-2 epidemic during January 2022 in England
Source: Nat Commun. 2022 Aug 3;13:4500. doi: 10.1038/s41467-022-32121-6 (PMC9349208; doi:10.1038/s41467-022-32121-6)
Supplement: Supplementary file 1 — Supplementary Information [file 41467_2022_32121_MOESM1_ESM.pdf]

Supplementary information:

Dynamics of a national Omicron SARS-CoV-2 epidemic during January 2022 in England

Paul Elliott[1,2,3,4,5,6, ‡,**], Oliver Eales[1,7,*], Barbara Bodinier[1,2,*], David Tang[1,2*], Haowei Wang[1,7], Jakob Jonnerby[1,8], David Haw[1,7], Joshua Elliott[3,9], Matthew Whitaker[1,2], Caroline E. Walters[1,7], Christina Atchison[1,3], Peter J. Diggle[10], Andrew J. Page[11], Alexander J. Trotter[11], Deborah Ashby[1], Wendy Barclay[9], Graham Taylor[9], Helen Ward[1,3,4,7], Ara Darzi[3,4,12], Graham S. Cooke [3,4,9], Marc Chadeau-Hyam[1,2,‡,**], Christl A. Donnelly[1,7,13,‡,**]

[1] School of Public Health, Imperial College London, UK

[2] MRC Centre for Environment and Health, School of Public Health, Imperial College London, UK

[3] Imperial College Healthcare NHS Trust, UK

[4] National Institute for Health Research Imperial Biomedical Research Centre, UK

[5] Health Data Research (HDR) UK, Imperial College London, UK

[6] UK Dementia Research Institute, Imperial College London, UK

[7] MRC Centre for Global infectious Disease Analysis and Jameel Institute, Imperial College London, UK

[8] National Heart and Lung Institute, Imperial College Healthcare NHS Trust, UK

[9] Department of Infectious Disease, Imperial College London, UK

[10] CHICAS, Lancaster Medical School, Lancaster University, UK and Health Data Research, UK

[11] Quadram Institute, Norwich, UK

[12] Institute of Global Health Innovation, Imperial College London, UK

[13] Department of Statistics, University of Oxford, UK

* These authors contributed equally

‡ These authors jointly supervised this work

**Corresponding authors: Paul Elliott, Marc Chadeau-Hyam, and Christl A Donnelly, p.elliott@imperial.ac.uk, m.chadeau@imperial.ac.uk, c.donnelly@imperial.ac.uk School of Public Health, Imperial College London, Norfolk Place, London, W2 1PG

Table S1. Unweighted and weighted prevalence of SARS-CoV-2 swab-positivity from REACT-1 across rounds 1 to 17. Weighted prevalence is based on round-specific weights calculated using the random iterative method (rim) approach.

Round	Tested swabs	Positive swabs	Unweighted prevalence (95% CI)	Weighted prevalence (95% CI)	First sample	Last sample
1	120,620	159	0.13% (0.11%, 0.15%)	0.16% (0.13%, 0.19%)	01/05/20	01/06/20
2	159,199	123	0.08% (0.07%, 0.09%)	0.09% (0.07%, 0.11%)	19/06/20	07/07/20
3	162,821	54	0.03% (0.03%, 0.04%)	0.04% (0.03%, 0.05%)	24/07/20	11/08/20
4	154,325	137	0.09% (0.08%, 0.11%)	0.13% (0.01%, 0.15%)	20/08/20	08/09/20
5	174,949	824	0.47% (0.44%, 0.50%)	0.60% (0.55%, 0.71%)	18/09/20	05/10/20
6	160,175	1,732	1.08% (1.03%, 1.13%)	1.30% (1.21%, 1.39%)	16/10/20	02/11/20
7	168,181	1,299	0.77% (0.73%, 0.82%)	0.94% (0.87%, 1.01%)	13/11/20	03/12/20
8	167,642	2,282	1.36% (1.31%, 1.42%)	1.57% (1.49%, 1.66%)	06/01/21	22/01/21
9	165,456	689	0.42% (0.39%, 0.45%)	0.49% (0.44%, 0.55%)	04/02/21	23/02/21
10	140,844	227	0.16% (0.14%, 0.18%)	0.20% (0.17%, 0.23%)	11/03/21	30/03/21
11	127,408	115	0.09% (0.07%, 0.11%)	0.10% (0.08%, 0.13%)	15/04/21	03/05/21
12*	108,911	135	0.12% (0.10%, 0.15%)	0.15% (0.12%, 0.18%)	20/05/21	07/06/21
13	98,233	527	0.54% (0.49%, 0.58%)	0.63% (0.57%, 0.69%)	24/06/21	12/07/21
14**	100,527	764	0.76% (0.71%, 0.82%)	0.83% (0.76%, 0.89%)	09/09/21	27/09/21
15***	100,112	1,399	1.40% (1.33%, 1.47%)	1.57% (1.48%, 1.66%)	19/10/21	05/11/21
16****	97,089	1,192	1.23% (1.16%, 1.30%)	1.41% (1.33%, 1.51%)	23/11/21	14/12/21
17†	102,174	4,073	3.99% (3.87%, 4.11%)	4.41% (4.25%, 4.56%)	05/01/22	20/01/22

* Sampling strategy changed for round 12 and subsequent rounds. Therefore unweighted prevalence is not directly comparable with previous rounds

** Including N=509 samples from 28-30 September 2021. Sample handling changed in round 14. Therefore prevalence is not directly comparable with previous rounds

*** Including N=93 samples (all negatives) from 6-8 November 2021, and N=86 samples with no collection/arrival dates

**** Including N=661 samples (including 12 positives) from 15-17 December 2021

† Including N=862 (including 36 positives) from 21-24 January 2022

Table S2. Table of growth rates per day (r), reproduction numbers (R) and doubling/halving times (in days) of SARS-CoV-2 swab-positivity from exponential model fits on data from round 17 (January 5 to 20, 2022)[1]

Rounds				Growth rate per day (r)	Reproduction number (R)*	Probability R>1, r>0	Doubling (+) / Halving (-) time (in days)
17	All positives			-0.015 (-0.022 , -0.008)	0.95 (0.93 , 0.97)	<0.01	-46.7 (-31.2 , -90.8)
		Age	Aged 17 and under	0.040 (0.026 , 0.054)	1.13 (1.09 , 1.18)	>0.99	17.3 (26.5 , 12.9)
			Aged 18 to 54	-0.045 (-0.055 , -0.035)	0.85 (0.82 , 0.89)	<0.01	-15.5 (-12.6 , -20.1)
			Aged 55 and over	-0.032 (-0.047 , -0.016)	0.89 (0.84 , 0.95)	<0.01	-21.7 (-14.6 , -42.1)
		Region	East Midlands	-0.009 (-0.035 , 0.017)	0.97 (0.88 , 1.06)	0.26	-79.7 (-19.7 , 40.3)
			West Midlands	0.004 (-0.016 , 0.024)	1.01 (0.95 , 1.08)	0.66	161.2 (-43.1 , 28.4)
			East of England	-0.008 (-0.033 , 0.016)	0.97 (0.89 , 1.05)	0.26	-84.3 (-21.0 , 42.4)
			London	-0.014 (-0.031 , 0.003)	0.96 (0.90 , 1.01)	0.06	-51.1 (-22.5 , 198.3)
			North West	-0.034 (-0.053 , -0.016)	0.89 (0.82 , 0.95)	<0.01	-20.2 (-13.0 , -44.2)
			North East	-0.008 (-0.036 , 0.018)	0.97 (0.88 , 1.06)	0.27	-82.9 (-19.4 , 38.1)
			South East	-0.009 (-0.030 , 0.012)	0.97 (0.90 , 1.04)	0.21	-79.4 (-23.4 , 58.5)
			South West	-0.003 (-0.030 , 0.024)	0.99 (0.90 , 1.08)	0.41	-218.4 (-22.8 , 29.0)
			Yorkshire and The Humber	-0.046 (-0.068 , -0.025)	0.85 (0.77 , 0.92)	<0.01	-15.1 (-10.2 , -28.1)

* Within-round R was calculated assuming an Omicron-specific Gamma-distributed generation time with mean 3.3 days and standard deviation of 3.5 days

[1] N=862 (including 36 positives) from January 21-24, 2022

Table S3A. Weighted prevalence of SARS-CoV-2 swab-positivity in round 16 and round 17 by sex, age, region, urban/rural area, employment type, and ethnic group. Weighted prevalence is based on round-specific weights calculated using the random iterative method (rim) approach.

		Round 16			Round 17		
Variable		Positive	Total	Weighted Prevalence	Positive	Total	Weighted Prevalence
Sex	Male	564	43,938	1.49% (1.36%, 1.64%)	1,858	45,031	4.52% (4.29%, 4.76%)
	Female	628	53,147	1.34% (1.23%, 1.46%)	2,215	57,141	4.30% (4.11%, 4.50%)
	Unknown	0	4	*	0	2	*
Age	05-11	236	4,811	4.74% (4.15%, 5.40%)	396	5,287	7.85% (7.10%, 8.69%)
	12-17	119	5,235	2.31% (1.91%, 2.80%)	255	5,351	5.20% (4.57%, 5.92%)
	18-24	20	2,093	0.93% (0.57%, 1.51%)	132	2,649	4.96% (4.11%, 5.96%)
	25-34	89	6,961	1.38% (1.10%, 1.74%)	392	7,683	5.08% (4.57%, 5.65%)
	35-44	205	11,925	1.71% (1.48%, 1.98%)	599	12,348	5.02% (4.62%, 5.46%)
	45-54	219	16,302	1.32% (1.15%, 1.52%)	678	16,996	4.05% (3.74%, 4.38%)
	55-64	188	20,905	0.97% (0.84%, 1.13%)	777	21,590	3.71% (3.45%, 3.99%)
	65-74	94	19,353	0.48% (0.39%, 0.59%)	597	20,088	3.06% (2.82%, 3.33%)
	75+	22	9,504	0.21% (0.13%, 0.32%)	247	10,182	2.46% (2.16%, 2.80%)
Region	South East	228	16,908	1.54% (1.33%, 1.77%)	545	18,287	3.23% (2.94%, 3.55%)
	North East	43	4,289	1.00% (0.70%, 1.42%)	273	4,541	6.86% (5.99%, 7.84%)
	North West	107	11,352	1.08% (0.86%, 1.35%)	589	11,867	5.36% (4.88%, 5.88%)
	Yorkshire and The Humber	96	9,261	1.32% (1.05%, 1.66%)	488	9,736	5.53% (4.98%, 6.13%)
	East Midlands	125	8,584	1.73% (1.42%, 2.11%)	352	9,045	4.15% (3.69%, 4.67%)
	West Midlands	89	9,715	1.03% (0.81%, 1.31%)	476	10,297	5.20% (4.70%, 5.75%)
	East of England	121	11,437	1.22% (0.99%, 1.49%)	350	11,938	3.42% (3.05%, 3.83%)
	London	239	14,819	1.84% (1.59%, 2.12%)	682	14,849	4.89% (4.49%, 5.31%)
	South West	144	10,724	1.57% (1.30%, 1.90%)	318	11,614	2.92% (2.58%, 3.30%)
Living in urban area	Yes	956	75,982	1.43% (1.33%, 1.54%)	3,402	79,566	4.72% (4.54%, 4.90%)
	No	233	20,951	1.35% (1.17%, 1.55%)	661	22,405	3.14% (2.89%, 3.42%)
	Unknown	3	156	2.25% (0.70%, 6.98%)	10	203	6.07% (2.99%, 11.92%)
Employment type	Health care or care home worker	92	7,694	1.41% (1.13%, 1.76%)	379	7,966	5.31% (4.75%, 5.94%)
	Other essential/key worker	247	13,427	1.96% (1.70%, 2.25%)	722	14,170	5.39% (4.97%, 5.84%)
	Other worker	525	38,338	1.52% (1.38%, 1.68%)	1,586	38,328	4.57% (4.32%, 4.83%)
	Not full-time, part-time, or self-employed	303	35,679	1.06% (0.92%, 1.21%)	1,281	39,792	3.52% (3.30%, 3.75%)
	Unknown	25	1,951	1.19% (0.77%, 1.83%)	105	1,918	5.70% (4.63%, 7.00%)
Ethnic group	White	1,003	85,420	1.35% (1.26%, 1.45%)	3,421	89,773	4.13% (3.98%, 4.28%)
	Asian	66	4,874	1.64% (1.24%, 2.17%)	297	5,383	6.58% (5.76%, 7.50%)
	Black	38	1,833	2.10% (1.48%, 2.98%)	105	1,730	6.55% (5.33%, 8.01%)
	Mixed	31	1,600	2.03% (1.40%, 2.94%)	91	1,745	5.56% (4.48%, 6.88%)
	Other	16	984	1.81% (1.08%, 3.01%)	58	963	7.35% (5.57%, 9.63%)
	Unknown	38	2,378	1.96% (1.40%, 2.74%)	101	2,580	4.32% (3.48%, 5.34%)

* Prevalence estimates are not reported if based on less than 5 observations

Table S3B. Weighted prevalence of SARS-CoV-2 swab-positivity in round 16 and round 17 by household size, number of children in the household, contact with a COVID-19 case, reported previous COVID-19, protective behaviours, symptom status and neighbourhood deprivation. Weighted prevalence is based on round-specific weights calculated using the random iterative method (rim) approach.

		Round 16			Round 17		
Variable		Positive	Total	Weighted Prevalence	Positive	Total	Weighted Prevalence
Household size	1	122	16,657	0.88% (0.72%, 1.09%)	536	17,601	3.15% (2.86%, 3.48%)
	2	287	39,443	0.77% (0.68%, 0.88%)	1,304	40,719	3.38% (3.19%, 3.59%)
	3	226	16,626	1.40% (1.22%, 1.62%)	786	17,384	4.95% (4.58%, 5.36%)
	4	362	17,026	2.29% (2.04%, 2.57%)	941	18,422	5.41% (5.04%, 5.80%)
	5	137	5,206	2.73% (2.25%, 3.32%)	337	5,719	6.23% (5.52%, 7.01%)
	6+	58	2,131	2.65% (2.00%, 3.50%)	169	2,329	7.72% (6.47%, 9.18%)
Number of children in the household	0	486	65,184	0.85% (0.76%, 0.95%)	2,306	68,713	3.57% (3.41%, 3.74%)
	1+	621	26,709	2.43% (2.23%, 2.65%)	1,535	28,206	5.92% (5.60%, 6.25%)
	Unknown	85	5,196	1.69% (1.35%, 2.12%)	232	5,255	4.75% (4.14%, 5.45%)
COVID case contact	No	529	75,990	0.81% (0.73%, 0.89%)	1,605	75,859	2.40% (2.27%, 2.54%)
	Yes, contact with a confirmed/tested COVID-19 case	463	6,048	8.00% (7.25%, 8.82%)	1,781	13,692	12.83% (12.20%, 13.49%)
	Yes, contact with a suspected COVID-19 case	51	1,523	3.29% (2.40%, 4.51%)	230	2,562	9.38% (8.14%, 10.79%)
	Unknown	149	13,528	1.25% (1.04%, 1.49%)	457	10,061	5.30% (4.78%, 5.86%)
Previous COVID-19	No	390	67,002	0.69% (0.62%, 0.77%)	1,010	67,800	1.75% (1.64%, 1.88%)
	Yes, confirmed by a test	577	9,173	6.60% (6.02%, 7.22%)	2,334	16,199	13.99% (13.39%, 14.61%)
	Yes, suspected	74	7,365	1.18% (0.91%, 1.52%)	269	8,091	3.84% (3.32%, 4.43%)
	Unknown	151	13,549	1.26% (1.05%, 1.51%)	460	10,084	5.32% (4.80%, 5.89%)
Shielding	Yes	117	8,963	1.53% (1.24%, 1.90%)	2,809	26,223	3.44% (3.18%, 3.73%)
	No	922	74,480	1.43% (1.33%, 1.54%)	801	65,800	4.60% (4.41%, 4.80%)
	Unknown	153	13,646	1.26% (1.05%, 1.51%)	463	10,151	5.31% (4.80%, 5.88%)
Frequency wearing mask indoors	Always	300	32,032	1.05% (0.92%, 1.19%)	1,774	49,795	3.84% (3.64%, 4.05%)
	Sometimes	469	36,533	1.47% (1.33%, 1.64%)	1,295	31,768	4.33% (4.07%, 4.61%)
	Hardly ever	39	3,272	1.15% (0.82%, 1.61%)	131	2,695	5.22% (4.32%, 6.30%)
	Never	10	1,305	0.62% (0.31%, 1.23%)	68	1,640	4.60% (3.43%, 6.15%)
	Unknown	374	23,947	1.80% (1.61%, 2.01%)	805	16,276	5.77% (5.35%, 6.22%)
Symptom status	Classic COVID symptoms*	525	7,818	6.96% (6.32%, 7.67%)	1,673	10,481	15.85% (15.06%, 16.68%)
	Other symptoms	211	15,377	1.52% (1.31%, 1.76%)	930	16,525	5.81% (5.40%, 6.24%)
	No symptoms	308	60,413	0.62% (0.55%, 0.70%)	1,019	65,149	1.87% (1.74%, 2.01%)
	Unknown	148	13,481	1.24% (1.03%, 1.49%)	451	10,019	5.26% (4.74%, 5.82%)
Deprivation	1 Most deprived	138	11,127	1.47% (1.21%, 1.78%)	639	11,245	6.08% (5.58%, 6.61%)
	2	245	16,263	1.61% (1.40%, 1.84%)	763	17,276	4.62% (4.27%, 4.99%)
	3	252	20,455	1.39% (1.21%, 1.58%)	831	21,472	3.96% (3.68%, 4.26%)
	4	255	23,307	1.26% (1.10%, 1.44%)	895	24,536	3.93% (3.66%, 4.22%)
	5 Least deprived	302	25,937	1.37% (1.21%, 1.54%)	945	27,645	3.69% (3.44%, 3.96%)

* Classic COVID symptoms: loss or change of sense of smell or taste, fever, new persistent cough

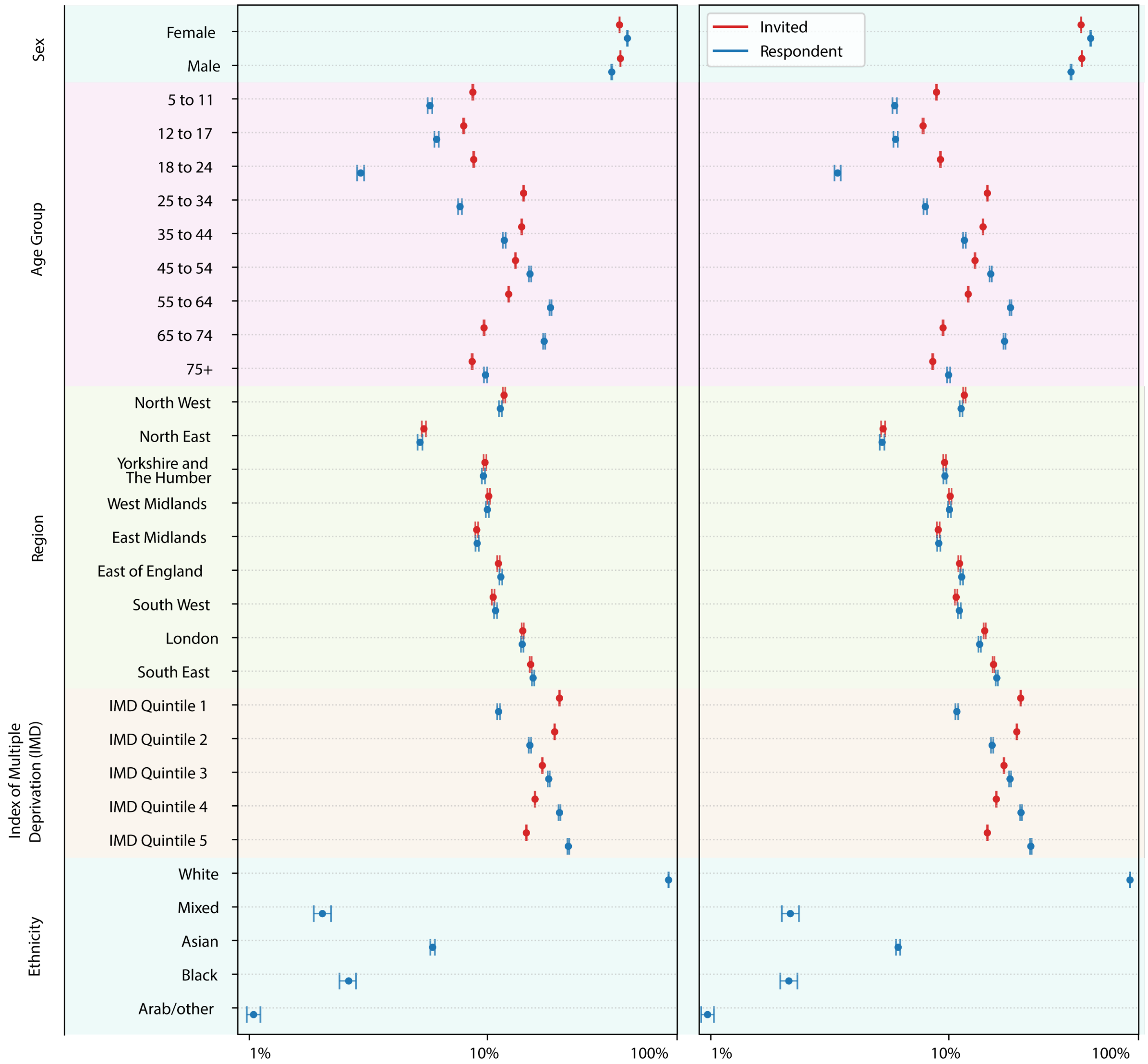

Figure S1. Proportion of individuals who were (i) invited (red) and (ii) accepted (blue) to participate in the REACT-1 study by sex, age, region, quintiles of Index of Multiple Deprivation (quintile 1 is the most deprived), and ethnicity[2]. Results are presented for round 16 (left) and round 17 (right).

[2] Data on ethnicity was not available for invited individuals who did not participate in the study.

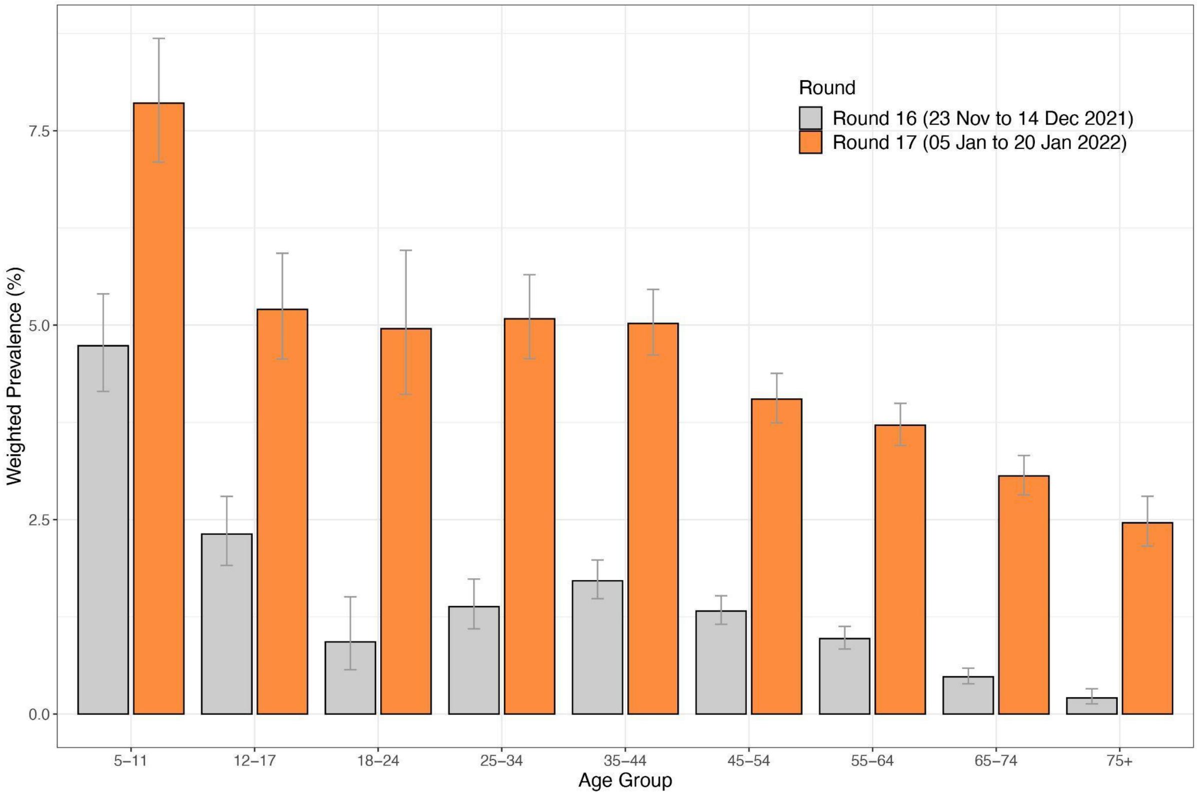

Figure S2. Weighted prevalence of SARS-CoV-2 swab-positivity by age group for round 16[3] and round 17[4]. Bars show the weighted prevalence point estimates (grey for round 16 and orange for round 17), and the vertical lines represent the 95% credible intervals.

[3] Includes N=661 samples (12 positives) obtained from December 15-17, 2021

[4] Includes N=862 samples (36 positives) from January 21-24, 2022

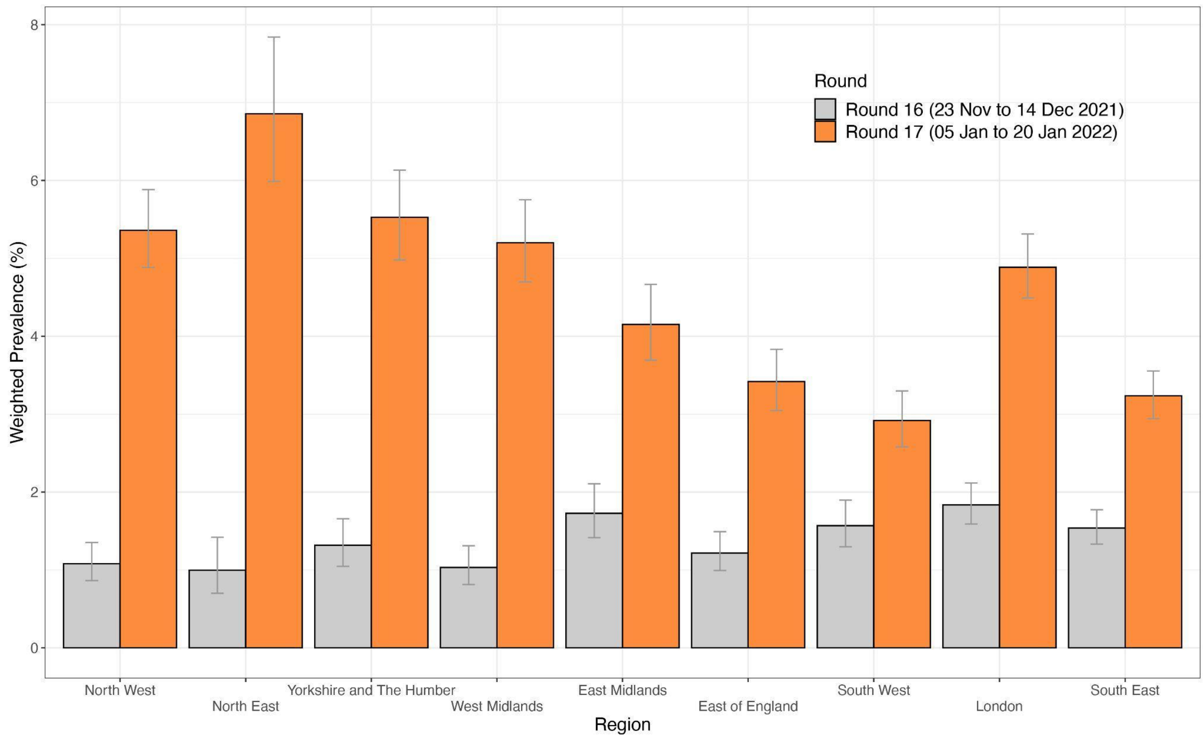

Figure S3 Weighted prevalence of SARS-CoV-2 swab-positivity by region or round 16[5] and round 17[6]. Bars show the weighted prevalence point estimates (grey for round 16 and orange for round 17), and the vertical lines represent the 95% credible intervals.

[5] Includes N=661 samples (12 positives) obtained from December 15-17, 2021

[6] Includes N=862 samples (36 positives) from January 21-24, 2022
