# Supplementary material for: Dynamics of a national Omicron SARS-CoV-2 epidemic during January 2022 in England
Source: Nat Commun. 2022 Aug 3;13:4500. doi: 10.1038/s41467-022-32121-6 (PMC9349208; doi:10.1038/s41467-022-32121-6)
Supplement: Supplementary file 2 — Description of Additional Supplementary Files [file 41467_2022_32121_MOESM2_ESM.pdf]

Description of Additional Supplementary Files

File Name: Supplementary Data 1
Description: ENA and GISAID accession numbers for sequencing data of the positive samples.
