# Supplementary material for: Dynamics of a national Omicron SARS-CoV-2 epidemic during January 2022 in England
Source: Nat Commun. 2022 Aug 3;13:4500. doi: 10.1038/s41467-022-32121-6 (PMC9349208; doi:10.1038/s41467-022-32121-6)
Supplement: Supplementary file 4 — Reporting Summary [file 41467_2022_32121_MOESM4_ESM.pdf]

nature portfolio

Corresponding author(s): Marc Chadeau-Hyam, Paul Elliott, Christl Donnelly

Last updated by author(s): May 26, 2022

Reporting Summary

Nature Portfolio wishes to improve the reproducibility of the work that we publish. This form provides structure for consistency and transparency in reporting. For further information on Nature Portfolio policies, see our Editorial Policies and the Editorial Policy Checklist.

Statistics

For all statistical analyses, confirm that the following items are present in the figure legend, table legend, main text, or Methods section.

n/a	Confirmed	
☐	☒	The exact sample size (*n*) for each experimental group/condition, given as a discrete number and unit of measurement
☐	☒	A statement on whether measurements were taken from distinct samples or whether the same sample was measured repeatedly
☐	☒	The statistical test(s) used AND whether they are one- or two-sided<br>*Only common tests should be described solely by name; describe more complex techniques in the Methods section.*
☐	☒	A description of all covariates tested
☐	☒	A description of any assumptions or corrections, such as tests of normality and adjustment for multiple comparisons
☐	☒	A full description of the statistical parameters including central tendency (e.g. means) or other basic estimates (e.g. regression coefficient) AND variation (e.g. standard deviation) or associated estimates of uncertainty (e.g. confidence intervals)
☐	☒	For null hypothesis testing, the test statistic (e.g. *F*, *t*, *r*) with confidence intervals, effect sizes, degrees of freedom and *P* value noted<br>*Give P values as exact values whenever suitable.*
☐	☒	For Bayesian analysis, information on the choice of priors and Markov chain Monte Carlo settings
☒	☐	For hierarchical and complex designs, identification of the appropriate level for tests and full reporting of outcomes
☒	☐	Estimates of effect sizes (e.g. Cohen's *d*, Pearson's *r*), indicating how they were calculated

Our web collection on statistics for biologists contains articles on many of the points above.

Software and code

Policy information about availability of computer code

Data collection	All data collection for the REACT1 study is captured with Questback (Spring 2020 installation).
Data analysis	Statistical analyses were all conducted using the R software version 4.0.5. Scripts used to run these analyses are available on the following GitHub Repository: https://github.com/mrc-ide/reactidd/tree/master/R/r17_scripts scripts (https://doi.org/10.5281/zenodo.6819880). Viral genome sequencing was done using the ARTIC protocol (version 4) for viral RNA amplification, CoronaHiT for preparation of sequencing libraries, the ARTIC bioinformatics pipeline (release 1.2.2.) and assigned lineages using PangoLEARN (version 2022-01-20).

For manuscripts utilizing custom algorithms or software that are central to the research but not yet described in published literature, software must be made available to editors and reviewers. We strongly encourage code deposition in a community repository (e.g. GitHub). See the Nature Portfolio guidelines for submitting code & software for further information.

Data

Policy information about availability of data

All manuscripts must include a data availability statement. This statement should provide the following information, where applicable:

- Accession codes, unique identifiers, or web links for publicly available datasets
- A description of any restrictions on data availability
- For clinical datasets or third party data, please ensure that the statement adheres to our policy

Access to REACT-1 individual-level data is restricted to protect participants' anonymity.

Summary statistics, descriptive tables from the current REACT-1 study are available at https://doi.org/10.5281/zenodo.6819880. REACT-1 study materials are available for each round at
https://www.imperial.ac.uk/medicine/research-and-impact/groups/react-study/react-1-study-materials/
Sequence read data are available without restriction from the European Nucleotide Archive at https://www.ebi.ac.uk/ena/browser/view/PRJEB37886, and consensus genome sequences are available from the Global initiative on sharing all influenza data (GISAID).
List of accession numbers is available as Supplementary Data 1.

Human research participants

Policy information about studies involving human research participants and Sex and Gender in Research.

Reporting on sex and gender	We only report results for sex, no information on gender has been collected. Results are presented for the whole study population and stratified by sex.
Population characteristics	Population characteristics we considered are: sex, residential location, age, ethnicity, occupation, household size an composition, protective behaviours, reported symptoms, and deprivation
Recruitment	We obtained a random population sample of adults in England, using the National Health Service (NHS) patient list, which includes name, address, age and sex of everyone registered with a general practitioner (and therefore is close to the entire UK population). We investigated differential response rates by age, sex, ethnicity and social factors. These were similar to those regularly observed in population surveys. In round 17, 12.2% of the invited participants returned swabs producing valid RT-PCR test results. Our descriptive analyses showed that those who agreed to participate were slightly older and from more affluent areas than the general population. We use weights, calculated for each participant in each round, to adjust for differential response rates in calculating prevalence estimates.
Ethics oversight	We obtained research ethics approval from the South Central-Berkshire B Research Ethics Committee (IRAS ID: 283787). Participants in the study (or their parents or guardians for children) provided informed consent.

Note that full information on the approval of the study protocol must also be provided in the manuscript.

Field-specific reporting

Please select the one below that is the best fit for your research. If you are not sure, read the appropriate sections before making your selection.

☐ Life sciences ☒ Behavioural & social sciences ☐ Ecological, evolutionary & environmental sciences

For a reference copy of the document with all sections, see nature.com/documents/nr-reporting-summary-flat.pdf

Behavioural & social sciences study design

All studies must disclose on these points even when the disclosure is negative.

Study description	REACT-1 is a series of community prevalence surveys including virological swabs and reverse transcriptase polymerase chain reaction (RT-PCR) tests from a series of age-sex stratified representative population samples of 100,000 to 150,000 individuals in England. The age range is 5 years and above.
Research sample	In each round of the (n=17) rounds of the study, individuals are sampled from the NHS patient list. In order to achieve the required sample size of 100,000 or 150,000 per rounds , up to 750,000 individuals aged 5 years and above were randomly selected and sent personalised invitations. For children (5 to 17 years old) the invitation is sent via parents/guardians. Conservative sample size calculations estimated that 150,000 and 100,000 participants would ensure the detection, at a 95% confidence level, of PCR positivity prevalence of 1.2 and 1.7%, respectively.<br>Total Population Round 17: N=102,174; 45,031 men 57,141 women aged 5 years and over. The sampled population was slightly more urban and affluent thant the general population of England, but we used rim weighting to correct for this.
Sampling strategy	Age and sex stratified representative samples of 100,000 to 150,000 were drawn from the general population of England. Initially we aimed to obtain approximately equal numbers of participants in each lower-tier local authority (LTLA) in England (N=315), but from round 12 (May 20 to June 7, 2021) we switched to obtaining a random sample in proportion to population size at LTLA level.
Data collection	Study participants were sent by post test kits and instructions. This included a self-administered throat and nasal swab, and completion of a short online or telephone questionnaire informing on demographic, behavioural factors and recent symptoms. A parent or guardian took the swab for children aged 12 years or below and helped completing the questionnaire. The swab was sent by courier (befor round 16), by courrer or post (in round 16) or post (in round 17) to a commercial laboratory. Participants were blinded to their RT-PCR test results while answering the questionnaire.
Timing	The sample collection of the REACT-1 study started in May 2020 and the seventeenth round was completed in January 2022. Round 17 data was collected from 5 to 24 January 2022.
Data exclusions	Participants with invalid RT-PCR test results were excluded (N=280, 385, and 105 in rounds 15, 16, and 17, respectively)

Non-participation	The 12.71 million registration letters sent in rounds 1 to 17 resulted in 3.13million (24.6%) registered participants, of whom, 70% successfully completed their test and follow-up questionnaires.
Randomization	Participants were not allocated to experimental groups

Reporting for specific materials, systems and methods

We require information from authors about some types of materials, experimental systems and methods used in many studies. Here, indicate whether each material, system or method listed is relevant to your study. If you are not sure if a list item applies to your research, read the appropriate section before selecting a response.

Materials & experimental systems

n/a	Involved in the study
☒	☐ Antibodies
☒	☐ Eukaryotic cell lines
☒	☐ Palaeontology and archaeology
☒	☐ Animals and other organisms
☒	☐ Clinical data
☒	☐ Dual use research of concern

Methods

n/a	Involved in the study
☒	☐ ChIP-seq
☒	☐ Flow cytometry
☒	☐ MRI-based neuroimaging
